# Is a contrast study really necessary prior to ureteroscopy?

**DOI:** 10.1590/1414-431X20154855

**Published:** 2015-11-17

**Authors:** O. Bayrak, A. Demirbas, O.G. Doluoglu, T. Karakan, B. Resorlu, S. Kardas, A. Tepeler, S. Tangal, S. Adanur, O. Celik

**Affiliations:** 1 Department of Urology, School of Medicine, Gazi University, Ankara, Turkey; 2 Department of Urology, Ankara Training and Research Hospital, Ankara, Turkey; 3 Department of Urology, Faculty of Medicine, Bezmialem University, Istanbul, Turkey; 4 Department of Urology, Faculty of Medicine, Ufuk University, Ankara, Turkey; 5 Department of Urology, Faculty of Medicine, Ataturk University, Erzurum, Turkey; 6 Department of Urology, Tepecik Training and Research Hospital, Izmir, Turkey

**Keywords:** Computed tomography, Imaging, Intravenous urography, Stone, Ureteroscopy

## Abstract

This study aimed to evaluate the effect of preoperative imaging techniques on the success and complication rates of ureteroscopy. We performed a retrospective analysis of 736 patients (455 males and 281 females), with a mean age of 45.5±15.2 years (range, 1-88 years), who underwent rigid ureteroscopic procedures for removal of ureteral stones. Patients were divided into 4 groups according to the type of imaging modality used: group I, intravenous urography (n=116); group II, computed tomography (n=381); group III, computed tomography and intravenous urography (n=91), and group IV, ultrasonography and abdominal plain film (n=148). Patients’ demographics, stone size and location, prior shock wave lithotripsy, lithotripsy technique, operation time, success rate, and rate of intraoperative complications were compared among the groups. There were no significant differences in success and complication rates among the groups. The stone-free rate after primary ureteroscopy was 87.1% in group I, 88.2% in group II, 96.7% in group III, and 89.9% in group IV (P=0.093). The overall incidence of intraoperative complications was 11.8%. According to the modified Satava classification system, 6.1% of patients had grade 1, 5.1% had grade 2, and 0.54% had grade 3 complications. Intraoperative complications developed in 12.1% of patients in group I, 12.6% of patients in group II, 7.7% of patients in group III, and 12.2% of patients in group IV (P=0.625). Our findings clearly demonstrate that ureteroscopic treatment of ureteral stones can be safely and effectively performed with no use of contrast study imaging, except in doubtful cases of anatomical abnormalities.

## Introduction

Non-contrast-enhanced computed tomography (NCCT) is considered the gold standard imaging technique in the diagnosis of urolithiasis because of its high sensitivity and specificity. Currently, patients who are admitted to hospital with acute flank pain are evaluated with NCCT (1,2). Patients with urinary stones are offered treatment options such as shock wave lithotripsy (SWL), ureteroscopy (URS), or percutaneous nephrolithotomy, or follow-up. Before interventions, contrast-enhanced re-imaging, such as intravenous urography (IVU) or computed tomography (CT), is recommended for evaluating anatomy of the renal collecting system (1). Despite this recommendation, most urologists do not perform any contrast-enhanced re-imaging before intervention after diagnosis of urinary stones. The most important reason for this lack of imaging is concern for future malignancies due to radiation exposure, and the risk for development of allergic reactions and contrast nephropathy due to use of contrast agents (3-7). Although low-dose CT has been recommended for decreasing radiation exposure, it is not used routinely in practice (3).

Because of all of these above-mentioned concerns, in this study, we aimed to investigate whether a contrast-enhanced study was necessary before an URS procedure in patients diagnosed with urinary tract calculi. We examined different imaging techniques to determine any differences for success and results of URS.

## Material and Methods

### Study population

We performed retrospective analysis of 736 patients (455 males and 281 females), with a mean age of 45.5±15.2 years (range, 1-88 years), who underwent rigid URS procedures for removal of ureteral stones. Cases requiring flexible URS were excluded from this study. Assessment of patients included the medical history, a physical examination, urinalysis, urine culture, complete blood count, bleeding and coagulation profile, serum biochemistry, and imaging modalities. The patients were divided into 4 groups according to the preoperative imaging modality used: patients who only had IVU (group I, n=116), those who only had NCCT (group II, n=381), those who had both NCCT and IVU (group III, n=91), and those who had ultrasonography and abdominal plain X-ray (group IV, n=148). Selection of the imaging modality was based on the surgeon's decision.

Patients’ demographics, size and location of stones, prior SWL, ureteral orifice dilation, preoperative stenting, lithotripsy technique, operation time, success rate, and intraoperative complications were documented and compared among the groups. The success of treatment was defined as the absence of residual stones after a single intervention. Stone-free status was assessed intraoperatively by direct URS, and postoperatively by imaging. Intraoperative complications were recorded according to the modified Satava classification system. Grade 1 complications included incidents without consequences for the patient, grade 2 complications were those that were treated intraoperatively with endoscopic surgery (grade 2a) or required endoscopic re-treatment (grade 2b), and grade 3 complications included incidents requiring open or laparoscopic surgery (8).

### Surgical technique

All URS procedures were performed under general or spinal anesthesia, in the lithotomy position. A semi-rigid ureteroscope was used in all patients, and a flexible-tipped guide wire was inserted through direct URS. Ureteral orifice dilation was performed in selected patients when the ureteroscope could not be easily advanced. The stones were fragmented with a pneumatic or holmium:YAG laser lithotripter until they were deemed small enough to pass spontaneously. Some small stones or residual fragments were removed with a basket catheter. Decision for placement of a double-J stent at the end of the procedure was based on the surgeon’s decision, and it was removed under local or brief anesthesia approximately 2 weeks later.

### Statistical analysis

Analysis of data was performed with SPSS for Windows, Version 15.0 (SPSS Inc., USA). Data are reported as means±SD. Kruskal-Wallis analysis of variance was used for intergroup comparisons of continuous variables (*post hoc*: Bonferroni), and the chi-square test was used for comparison of categorical variables. Statistical significance was set at P<0.05.

## Results

The study group consisted of 455 males and 281 females, with a mean age of 45.5±15.2 years (range, 1-88 years). The mean age was 45.4 years (18-81 years) in group I, 46.6 years (1-79 years) in group II, 45.9 years (8-77 years) in group III, and 42.8 years (4-76 years) in group IV, with a significant difference among the groups (P=0.009). The stones were located in the distal ureter in 67.6% of patients, the mid-ureter in 23.1% of patients, and the proximal ureter in 9.2% of patients. There was no difference in localization of stones among the groups (P=0.067). The mean stone size was 11.6±3.9 mm when all of the patients who were included in the study were considered. The mean stone size was 12.3 mm (5-30 mm), 11.3 mm (4-25 mm), 11.5 mm (3-23 mm), and 11.3 mm (4-30 mm) in groups I, II, III, and IV, respectively (P=0.122). SWL was performed in 24.9% of the patients before surgery, and this rate was 22.4% in group I, 25.7% in group II, 23.1% in group III, and 25.7% in group IV (P=0.866). Table 1 shows the characteristics of patients and stones.

**Table:**
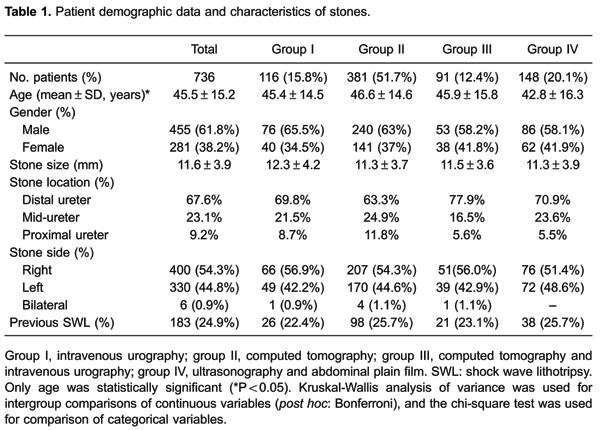

Ureteral orifice dilation was performed in 19.8% of the patients. The rate of ureteral orifice dilation was significantly different among the groups (P<0.001). The mean duration of surgery was 35.8±13.1 min and was also significantly different among the groups (P=0.026). There were no differences among the groups for the rate of success or complications. After one URS session, 658 (89.4%) patients were stone-free (among groups, P=0.093). The intraoperative rate of complications was 11.8% for all of the patients included in the study. According to Satava classification, 6.1% of the complications were grade 1, 5.1% were grade 2, and 0.54% were grade 3. The intraoperative rate of complications was not significantly different among groups (P=0.630). Table 2 shows procedural characteristics and Table 3 shows the clinical data in relation to complications.

**Table:**
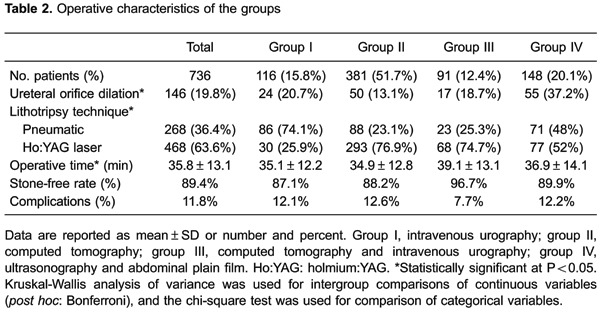

**Table:**
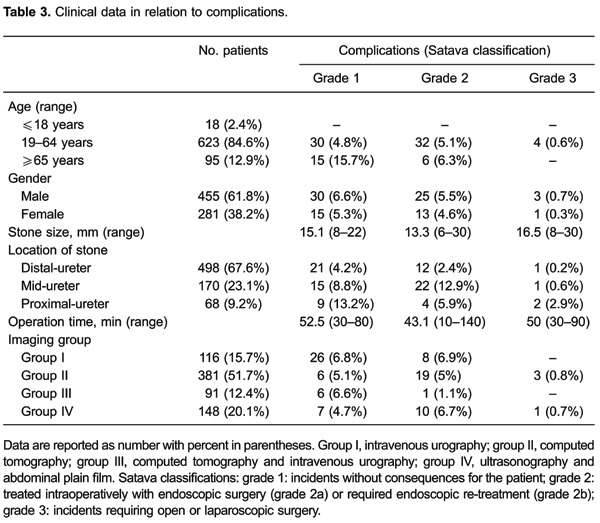

## Discussion

Radiological imaging techniques constitute the most important step in diagnosis and treatment planning of urinary stone disease. Currently, the preference and timing of imaging techniques are controversial in the diagnosis and follow-up of this disease. Current European Association of Urology guidelines recommend NCCT as the gold standard imaging technique in patients who are admitted with acute flank pain (1). The advantages of NCCT over other imaging techniques include its high sensitivity and specificity, and its ability to determine stones, other pathologies, the distance between the stone and skin, urinary system anatomy, and density of mass, as well as absence of any need for any preparation or contrast agent (9).

High radiation exposure is the most important disadvantage of NCCT. Furthermore, many patients with flank pain may be admitted to the emergency department with similar complaints repeatedly, and may need recurrent CT imaging (10). This is why European Association of Urology guidelines recommend low-dose CT in the diagnosis of urinary stones, but this is not followed routinely in practice (1). Miglioretti et al. (3) investigated radiation exposure due to abdominal/pelvis CT in children. They reported that the mean radiation dose exposed per CT was 10.6 mSv in children younger than 5 years of age, and 14.8 mSv in children aged between 10-15 years. The authors reported that those doses were much greater than the doses recommended for low-dose CT (radiation exposure: 0.97-1.9 mSv). They estimated that one per 300-390 girls and one per 670-760 boys would develop solid cancers in the future because of this exposure (3). Epidemiological studies have reported that radiation exposure during one CT is similar to the amount of radiation exposure in Nagasaki and Hiroshima after atomic bombs (4-7).

An additional radiological imaging before URS increases radiation exposure, and may increase the risk for severe allergic reactions and contrast nephropathy because of use of contrast agents. Mechanisms, such as direct toxicity, hemodynamic changes, and tubular obstruction, are thought to play role in the pathophysiology of acute renal failure due to radiocontrast agents (11). Radiocontrast agents are responsible for 5-10% of patients hospitalized for acute renal failure, and they are the most frequent agents that cause acute renal failure following aminoglycosides (12). Currently, there is no curative treatment for contrast nephropathy.

The prevalence of allergic reactions due to contrast agents has been reported as 2-8%, and they cause severe, life-threatening reactions in 0.1% of cases (13,14). The rate of mortality due to contrast agent use during IVU procedures is 0.01-0.001% (13,15). Although allergic reactions and nephrotoxicity related to contrast agents are rare, routine use of contrast-enhanced radiological imaging modalities in every patient would increase the number of patients affected. In addition, bowel preparation before IVU and a long duration of procedure are quite disturbing for patients.

In our study, contrast-enhanced radiological imaging was performed in 207 (28.1%) patients, but we do not know the prevalence of nephrotoxicity or allergic reactions. This is the most important limitation of our study. The reason for this lack of knowledge is the retrospective and multicenter design of our study. We did not find any differences in the rate of success or complications after surgery between patients who had direct X-rays and contrast-enhanced imaging modalities preoperatively. Therefore, this raises the question whether contrast-enhanced imaging modalities, which have many potential risks, are really necessary for those patients.

Currently, there is no consensus on the preference and timing of the imaging techniques used in the diagnosis and follow-up of urinary stone disease (3,7,8,16,17). Therefore, prospective, randomized studies are needed to measure the amount of radiation exposure, evaluate the adverse effects due to contrast agents, and patients’ satisfaction. Experimental studies are required to analyze injury related to these imaging modalities. However, our study is important because it is the first clinical study to draw attention to this topic, despite all of its limitations.

Low-dose NCCT is a reliable and safe imaging modality for diagnosis of urinary stones and evaluation of patients before URS. In addition to radiation exposure, use of extra imaging modalities in the preoperative period carries the risk for nephrotoxicity and allergic reactions. Therefore, we believe that contrast-enhanced imaging modalities should not be used routinely in the preoperative period, except for patients with suspicion of urinary tract abnormalities (e.g., horseshoe kidney and pelvic ectopic kidney) or obstruction (e.g., ureteropelvic or ureterovesical obstruction and ureteral stricture) in ultrasonography or NCCT. Retrograde pyelography can be performed during URS as an alternative to prior contrast-enhanced radiological exams that could avoid high radiation exposure and allergic reactions.
